# Toxicological Assessment of β-(1→6)-Glucan (Lasiodiplodan) in Mice during a 28-Day Feeding Study by Gavage

**DOI:** 10.3390/molecules171214298

**Published:** 2012-12-03

**Authors:** Janaína A. Túrmina, Emerson Carraro, Mário A. Alves da Cunha, Robert F. H. Dekker, Aneli M. Barbosa, Fábio Seidel dos Santos, Luiz A. Silva, Carlos R. M. Malfatti

**Affiliations:** 1 Pharmaceutical Science and Biodiversity Postgraduate Programs, Midwest State University, Campus CEDETEG, Guarapuava, PR, 85040-080, Brazil; E-Mails: janainaangela@hotmail.com (J.A.T.); emersoncarraro@bol.com.br (E.C.); fabio_seidel@hotmail.com (F.S.S.); luizbiologia@hotmail.com (L.A.S.); 2 Department of Chemistry, Federal Technological University of Parana, Pato Branco, PR, 85503-390, Brazil, E-Mail: mcunha@utfpr.edu.br; 3 Biorefining Research Institute, Lakehead University, Thunder Bay, ON P7B 5E1, Canada; E-Mails: rdekker@lakeheadu.ca (R.F.H.D.); anelibarbosa@gmail.com (A.M.B.)

**Keywords:** *Lasiodiplodia theobromae* MMPI, fungal β-glucan, toxicity evaluation, Swiss albino mice

## Abstract

Studies evaluating the toxicity caused by fungal exopolysaccharides of the β-(1→6)-d-glucan type are rare. In this study, the toxicological effects of sub-chronic treatments with lasiodiplodan (β-(1→6)-d-glucan from *Lasiodiplodia theobromae* MMPI) were evaluated in mice through the assessment of biochemical, hematological, and histopathological alterations. Thirty-two mice (16 male, 16 female) were used in this study divided in two groups; one group received lasiodiplodan (50 mg/kg body weight) daily for 28 days *via* gavage, and another (control group) received saline during the same period. Blood samples were collected via cardiac puncture for hematological and biochemical analyses. Liver, heart, kidney, and spleen were collected for histopathological analysis. Statistical analysis was performed through one-way analysis of variance and only *p* < 0.05 F-values were presented. Significant reduction in blood glucose in the male group (35%; *p* < 0.01), transaminases activity in both sexes (AST and ALT; ~35%; *p* < 0.05), and urea (20%; *p* < 0.01) in the female group was observed with the lasiodiplodan treatment. The results showed that sub-chronic treatments with lasiodiplodan did not generate hematological and histopathological alterations leading to signs of toxicity in healthy mice, independent of gender.

## 1. Introduction

Non-cellulosic β-glucans have been considered as natural products that can be useful in preventing and treating several disease conditions in humans [1]. The β-glucans linked by (1→3;1→4)-d-glucosidic bonds found in oat, barley, and wheat bran are considered dietary fiber and reported to have the potential to reduce the risk of some chronic diseases related to the cardiovascular system, metabolic syndrome, type-2 diabetes, colon and breast cancers, obesity, and the gastrointestinal tract. These polysaccharides are involved in controlling blood levels of glucose, insulin, lipids, and blood pressure, which are associated with obesity, diabetes, and metabolic syndrome [2,3].

The content of β-glucans in cereals, albeit low, varies, and their extraction can be costly. Fungal β-glucans of the following type: β-(1→3)-, β-(1→3;1→6)-, and β-(1→6)-linked, on the other hand, can be extracted from the cell wall of yeasts and edible mushrooms, or they can be obtained by submerged fermentation where they are secreted as exopolysaccharides (EPS’s) in the culture medium [1]. The fungal β-glucans of the (1→3)- and (1→3;1→6)- linked types have similarly been demonstrated to have potential in treating diabetes and associated cardiovascular risks, microbial infections, Alzheimer’s disease, acquired immunodeficiency syndrome (AIDS), and multiple sclerosis [4,5]. The advantage of the fungal EPSs is that the production process can be optimized in bioreactors [6] and the products are easily obtained through precipitation from the culture medium with alcohol. Their production is thus considered more cost effective.

β-Glucans, pharmacologically classified as biological response modifiers (BRM) [7], are derived from the diet (cereals, mushrooms) and not synthesized by mammals. Mice studies originally showed them to stimulate the immune system [8]. The most studied fungal β-glucans possessing immunomodulatory effects have branched structures and a backbone chain made up of glucose residues linked by β-(1→3) bonds, to which, glucose residues of β-(1→6) bonds that form the side chains are linked. The molecular size, conformation, and degree of branching appear to be important determinants for the immune response, however, it is still unclear how this process occurs [1,6,9]. These β-glucans are reported as non genotoxic and capable to exert a protective effect that is dose dependent in mice [9]. Several of the β-glucans of the β-(1→3);(1→6) type also exhibit hypoglycaemic and hypocholesterolaemic effects in diabetes-induced and hyperlipidaemic conditioned rodents, respectively [10,11,12,13].

There are few reports on EPSs of the β-(1→6)-d-glucan type, which are considered rare. β-Glucans of this type were first isolated from species of lichens of the Umbilicariaceae family, from which they were extracted and known as pustulan [14]. Exocellular-produced β-(1→6)-d-glucans are described as produced by the fungi *Guignardia citricarpa* [15] and *Lasiodiplodia theobromae* [16]. The β-(1→6)-d-glucan from *L. theobromae* MMPI, named lasiodiplodan, presents anti-proliferative activity on MCF-7 breast cancer cells [17].

Investigations on the chronic and sub-chronic intake of β-glucans *in vivo*, in animal models that assess toxicological effects are scarce [18], and to the authors’ knowledge, there are no reports assessing the toxicity of β-(1→6)-d-glucans including lasiodiplodan. In the work presented here, the toxicity effects of lasiodiplodan at sub-chronic doses were assessed by examining various biochemical, hematological, and histopathological parameters producing alterations in metabolic activities, in different tissues in vital organs (heart, kidney, spleen, liver), of Swiss albino mice. We also report on blood glucose and lipids profiles after the administration of lasiodiplodan.

## 2. Results and Discussion

### 2.1. Biochemical Alterations Caused by Lasiodiplodan Intake

**Figure 1:**
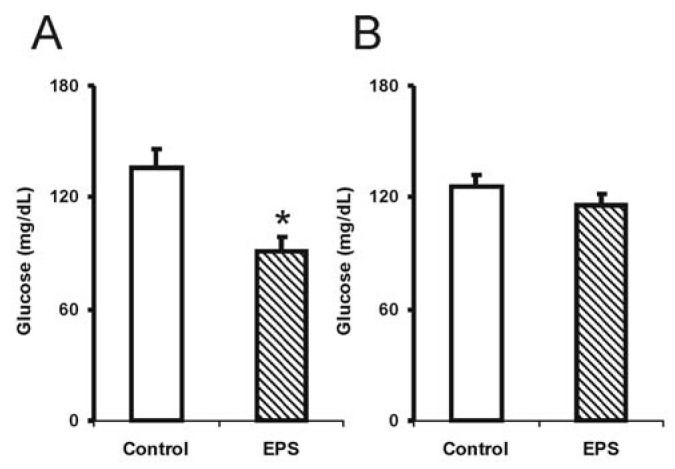
Levels of blood glucose in mice exposed to sub-chronic treatment with lasiodiplodan. (**A**) male and (**B**) female groups. (*) Values based on *p* < 0.05.

The sub-chronic use of lasiodiplodan (β-(1→6)-glucan) resulted in a significant reduction in blood glucose levels (35%) in the male group [F (1.14): 9.5; *p* < 0.01], while the tendency to reduce glucose (10%) was tenuous in the female group (Figure 1). The reason for this discrepancy is not known considering the health status of the mice used in this experiment.

Recently, Miranda-Nantes *et al.* [13] showed a hypoglycaemic and hypocholesterolaemic effect of a fungal β-glucan, botryosphaeran, a β-(1→3;1→6)-d-glucan produced by *Botryosphaeria rhodina*, which is a perfect form of *Lasiodiplodia theobromae*. This investigation was developed over two weeks in streptozotocin (STZ)-induced diabetic rats, resulting in a reduction in 52% in blood glucose levels and 27% reduction in LDL-cholesterol levels in hyperlipidaemic-conditioned rats. Soluble polysaccharides extracted from *Tremella mesenteric* also showed a significant effect in reducing glucose levels in STZ-induced hyperglycaemia rats and in animals used as genetic models for diabetes [10,19]. There are no studies reporting the effects of β-(1→6)-d-glucan on normal or diabetic animals.

**Figure 2:**
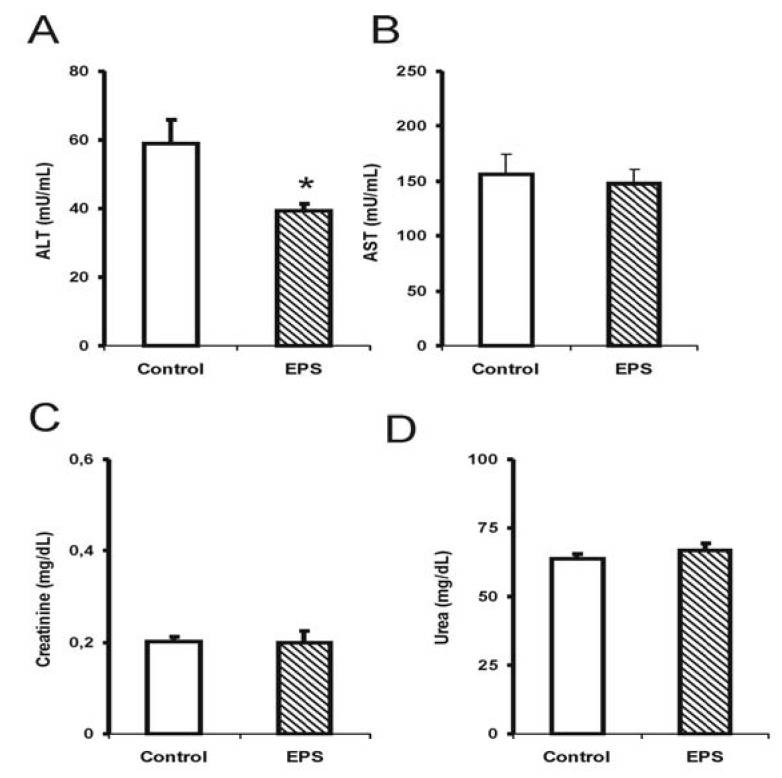
Hepatotoxicity markers in male mice submitted to sub-chronic treatment with lasiodiplodan. Specific transaminases (**A**) ALT and (**B**) AST; (**C**) creatinine, and urea (**D**). (*) Values based on *p* < 0.05.

**Figure 3:**
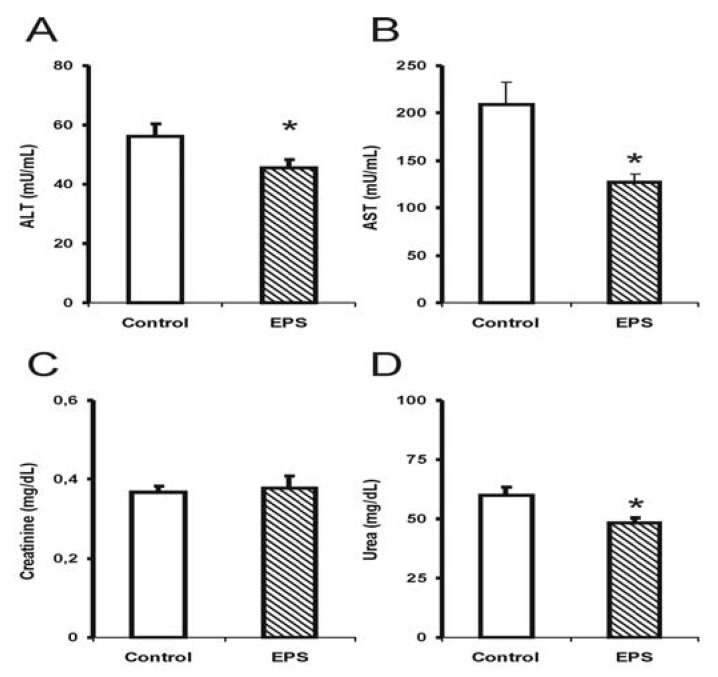
Hepatotoxicity markers in female mice submitted to sub-chronic treatment with lasiodiplodan. Specific transaminases (**A**) ALT and (**B**) AST; (**C**) creatinine, and urea (**D**). (*) Values based on *p* < 0.05.

Alterations in hepatotoxicity markers were observed through a significant reduction in transaminase activities; alanine transaminase (ALT) in males (35%) [F (1.14): 3.0; *p* < 0.05; see Figure 2] and females (20%) [F (1.14): 4.5; *p* < 0.05; see Figure 3], and a 40% reduction in aspartate transaminase (AST) activity for the female group [F (1.14): 9.9; *p* < 0.05; see Figure 3]. The creatinine and urea levels were similar in the male group although 20% reduction in the urea values in the female group was observed [F (1.14): 8.3; *p* < 0.01]. 

Few studies have investigated the effect of sub-chronic or chronic β-glucan treatments on biochemical markers and toxicity using animal models. The results obtained in this study showed that the sub-chronic treatment with β-(1→6)-glucan, lasiodiplodan in male and female mice did not elevate the AST and ALT levels suggesting the absence of liver damage. In another study, the EPS from *Lentinulla edodes* corroborated the findings from the present study [20]; this EPS also reduced ALT and AST levels.

The markers for renal injury, creatinine and urea, did not suggest alterations in renal function in the mice treated with lasiodiplodan compared to the control group. Increased levels of urea and creatinine could suggest renal function impairment; elevated levels were not observed in these parameters in this study. The urea levels in the female group were somewhat lower (20%). It was concluded that no toxicological significance was associated with the administration of lasiodiplodan in the kidneys in the healthy mice.

There is much interest in using natural products for the control of diabetes, and insulin resistance causing obesity, which has alarmingly increased worldwide (http://www.worlddiabetesfoundation.org). Thus, the use of natural biopolymers, and more specifically β-glucans, has shown promising results in glycaemic control [4,12,21]. The mechanism of action to explain the reduction in plasma glucose levels, and cholesterol levels in rats with hypercholesterolaemia, is still not well understood. Nevertheless, it is believed that many of these effects might be related to a “corrective” biochemical action in pancreatic and liver functions perceived by the reduction in hepatic transaminase activities (ALT and AST) as demonstrated by this study. The mechanism that specifically triggers this effect is still unknown. The study reported here evaluated the possible existence of some toxic effects from the sub-chronic use of β-(1→6)-glucan. Interestingly, a reduction in glucose levels (male mice group), and hepatic transaminase activity were observed in healthy mice. These findings could be taken into consideration for future investigations in the use of β-glucans as preventive and coadjuvant supporting treatments of different chronic-degenerative diseases such as diabetes, hypertension, and dyslipidemia.

In the present study, toxicity arising from biochemical, immunological and histopathological variables resulting from treatment with EPS in both sexes were not identified. However, differences in terms of responses in biochemical parameters were observed, where the males showed reduction in the activity of liver enzymes and blood glucose values when compared with females. Therefore, the differences in the pulse of growth hormone [22] and even in the gastrointestinal absorption rate of EPS, when comparing males and females, indicates a pathway that explain these results, given that the exact mechanism of gastrointestinal absorption of EPS and the subsequent hormonal responses still remain to be elucidated.

### 2.2. Lipid Profile Arising from Administering Lasiodiplodan to Mice

The values of total cholesterol, LDL-, VLDL-, and HDL- cholesterols, and triacylglycerols did not show statistically significant differences between the control and lasiodiplodan-treated groups in healthy mice from both genders (Table 1). The animals in this study did not present hypercholesterolaemia and hyperlipidaemia according to the reference values presented by males and females treated with saline in the control groups (see Table 1).

Several studies examined the hypocholesterolaemic effects of β-glucans on hyperlipidaemic rats using polysaccharides extracted from yeasts (cell wall β-(1→3;1→6)-glucans [11]) and plants (barley β-glucan [23]), however, relatively fewer studies reported on exopolysaccharides produced by submerged fermentation [12]. Thus, future studies using diabetic, hypercholesterolaemic, and hyperlipidaemic animal models could evaluate the potential of the preventive effects of β-(1→6)-glucans. Although the mechanism involved in hypocholesterolaemic activity of different β-glucans is presently unclear, it has been proposed that the increased viscosity of these biopolymers might decrease the absorption of cholesterol and triacylglycerols in the gut, or decrease the activity of digestive enzymes, thereby reducing the lipid levels [23].

### 2.3. Hematological Parameters

Differences in leukocyte counts between the lasiodiplodan-treated and control groups in both genders of healthy mice were not observed. Likewise, alterations in the differential leukocyte counts were not observed as shown in Table 2. Our results only suggest an absence of inflammatory response, given that the histological analysis does not present indicative signs of edema, necrosis, or cellular differentiation in according to previously reported in the literature [24].

### 2.4. Histopathological Parameters

**Figure 4:**
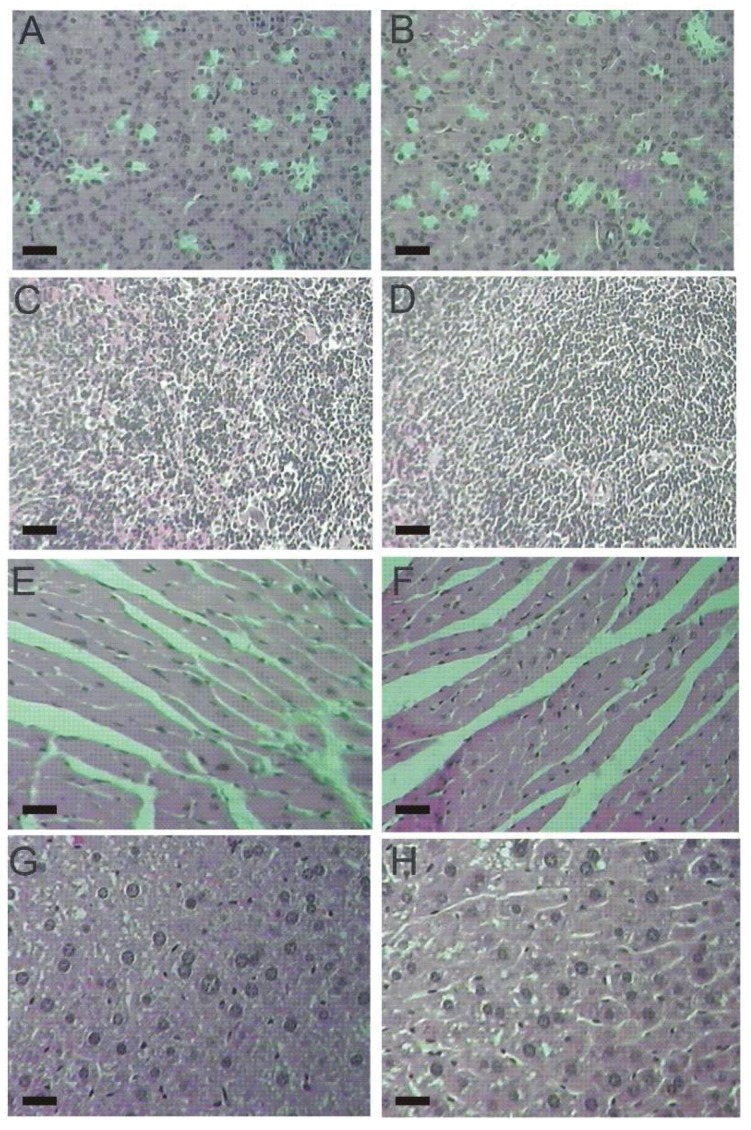
Histological tissue sections from male mice (100×). (**A**) kidney from the control group; (**B**) kidney from the lasiodiplodan-treated group; (**C**) spleen from the control group; (**D**) spleen from the lasiodiplodan-treated group; (**E**) heart from the control group; (**F**) heart from the lasiodiplodan-treated group; (**G**) hepatic tissue from the control group; and (**H**) hepatic tissue from the lasiodiplodan-treated group. Scale bar 25 micron.

**Figure 5:**
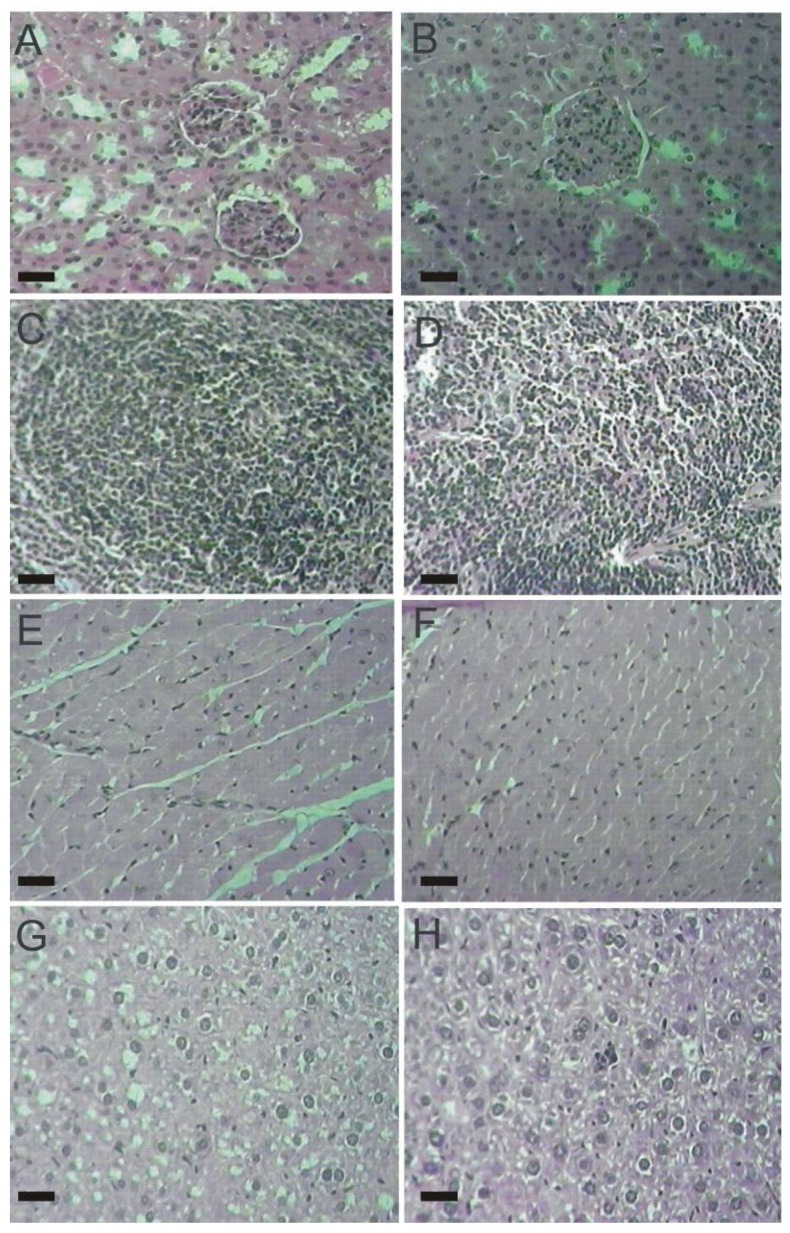
Histological tissue sections from female mice (100×). (**A**) kidney from the control group; (**B**) kidney from the lasiodiplodan-treated group; (**C**) spleen from the control group; (**D**) spleen from the lasiodiplodan-treated group; (**E**) heart from the control group; (**F**) heart from the lasiodiplodan-treated group; (**G**) hepatic tissue from the control group; and (**H**) hepatic tissue from the lasiodiplodan-treated group. Scale bar 25 micron.

The histopathological examination of vital organs (kidney, spleen, heart, liver) did not detect alterations between male and female mice from both, control and lasiodiplodan-treated groups (see Figure 4 and Figure 5). The use of lasiodiplodan did not promote histological changes in the kidneys in mice from both genders. The cortex presented evenly distributed glomeruli with spaces in addition to fine capillary tufts (Figure 4A,B, Figure 5A,B). The spleen presented normal histological characteristics (Figure 4C,D, Figure 5C,D); the cardiac tissue remained intact with no inflammatory infiltration, edema, or fibrillary degeneration (Figure 4E,F, Figure 5E,F). The absence of histopathological alterations in the liver (Figure 4G,H, Figure 5G,H) is in accordance with the biochemical profile presented by the animals in this study.

## 3. Experimental 

### 3.1. Microorganism and Cultivation 

*Lasiodiplodia theobromae* isolate MMPI was cultivated in nutrient medium containing mineral salts, glucose, and yeast extract for 96 h at 28 °C in a stirred-tank bioreactor according to Cunha *et al.* [17].

### 3.2. Recovery of Exopolysaccharide

Fungal biomass was separated from the fermentation broth by centrifugation (1,500 *×g*/30 min) after 96 h of cultivation. The supernatant was exhaustively dialyzed against distilled water over 48 h, exopolysaccharide was precipitated by the addition of four volumes of absolute ethanol, and the mixture incubated at 4 °C overnight. The precipitate was recovered by filtration, solubilized in water at 60 °C, and followed by dialysis against distilled water. The dialyzed exopolysaccharide portion was lyophilized to obtain dried lasiodiplodan.

### 3.3. Preparation of the Lasiodiplodan Stock Solution 

An EPS (lasiodiplodan) stock solution was prepared in isotonic saline solution at a concentration of 5 g/L, autoclaved at 121 °C for 20 min, and used for administration in mice by gavage.

### 3.4. Experimental Design

Thirty-two healthy Swiss albino mice (*Mus musculus*; 16 male, 16 female), with weights between 25 and 30 g and age ~60 days, were used in this study. The animals were kept in polyethylene cages and maintained under controlled conditions of temperature (25 °C) and light/dark cycle (12 h/12 h), with water and food (Purina chow) available *ad libitum*. The animals were divided into four groups of 8 mice each. One male and one female group were treated with lasiodiplodan, and the other two groups were used as controls. The control groups, male and female, received physiological saline daily, via gavage, during 28 days. The other two groups of mice were administered daily doses of 50 mg lasiodiplodan/kg body weight (b.w.) per animal, by gavage, during 28 days. The maximum dose concentration was chosen based on the solubility of EPS.

### 3.5. Biochemical and Hematological Analyses

At the end of the treatment period (28 days), the mice were fasted for 12 h and sacrificed by anesthetization using ketamine and xilazine; blood samples were collected through cardiac puncture. The blood samples were placed in heparinized tubes and immediately processed using BioLab commercial kits designed for the following biochemical determinations: glucose, total cholesterol, LDL-cholesterol, VLDL-cholesterol (Triglycerides/5), HDL-cholesterol, alanine transaminase (ALT), aspartate transaminase (AST), creatinine, and urea. Hematological parameters such as total white blood cells (WBC) were evaluated using a Neubauer chamber [25]. Blood smears were stained with Giemsa for differential WBC counts [26]. 

### 3.6. Histopathological Analysis 

The vital organs: liver, heart, kidneys, and spleen were removed following euthanasia and fixed in 10% formaldehyde for 48 h. These were sectioned using a microtome and stained with Hematoxylin-Eosin (H.E.). 

### 3.7. Statistical Analysis

The analyzed data was expressed as the mean ± SD (standard deviation). The statistical analysis was performed by a one-way analysis of variance (ANOVA) and F-values were presented only if *p* < 0.05. *Post-hoc* analysis was performed when appropriate using the Tukey’s test.

## 4. Conclusions

This study demonstrated that the intake of lasiodiplodan (β-(1→6)-d-glucan) did not produce signs of toxicity in mice, regardless of gender. The administration of lasiodiplodan by gavage at the concentration of 50 mg/kg b.w./day induced significant hypoglycaemic activity in male mice (blood glucose reduction of 35%) and a reduction in transaminase activity (ALT and AST) in male and female mice. Sub-chronic treatment with lasiodiplodan did not result in any hematological and histopathological alterations. Future studies will be directed towards the analysis of the antidiabetic properties of this β-(1→6)-d-glucan and its mechanism of action involved in modifying biochemical parameters. 

## Figures and Tables

**Table 1:** Blood lipid profile of male and female mice groups after a 28-day treatment with lasiodiplodan by gavage.

Treatment group ^a^	Cholesterol (mg/dL)	HDL ^b^ (mg/dL)	LDL ^c^ (mg/dL)	VLDL ^d^ (mg/dL)	Triacylglycerol (mg/dL)
*Male*					
Control	143 ± 14.2	37 ± 3.9	70 ± 14.0	36 ± 12.0	178 ± 61.0
Lasiodiplodan	133 ± 17.2	36 ± 9.0	61 ± 14.1	36 ± 10.0	180 ± 50.0
*Female*					
Control	100 ± 8.17	26 ± 4.8	42 ± 16.6	32 ± 12.4	161 ± 19.0
Lasiodiplodan	91 ± 17.11	20 ± 6.6	38 ± 16.7	33 ± 8.5	164 ± 64.6

^a^ n = 8; Cholesterols of: ^b^ high-density lipoprotein; ^c^ low-density lipoprotein; ^d^ very low-density lipoprotein (Triglycerides/5).

**Table 2:** Hematological parameters of male and female mice groups after a 28-day treatment with lasiodiplodan by gavage.

Leukogram	Control (Mean ± SD) ^a^		Lasiodiplodan (Mean ± SD) ^a^	
	Males	Females	Males	Females
Total leukocytes (mm^3^)	4.30 ± 0.20	4.00 ± 0.31	4.20 ± 0.12	4.10 ± 0.67
Neutrophils (%)	25.00 ± 1.58	25.50 ± 1.50	21.00 ± 2.21	21.87 ± 3.32
Basophils (%)	0.62 ± 0.18	1.12 ± 0.22	1.00 ± 0.18	1.42 ± 0.20
Lymphocytes (%)	71.12 ± 2.01	72.12 ± 2.30	75.50 ± 2.73	75.12 ± 3.18
Monocytes (%)	2.85 ± 0.91	2.95 ± 0.85	1.97 ± 0.45	3.00 ± 0.46
Eosinophils (%)	0.37 ± 0.18	0.75 ± 0.17	0.12 ± 0.08	0.12 ± 0.08

^a^ n = 8.

## References

[1] Novak M., Vetvicka V. (2008). β-Glucans, history, and the present: Immunomodulatory aspects and mechanism of action. J. Immunotoxicol..

[2] El Khoury D., Cuda C., Luhovyy B.L., Anderson G.H. (2012). Beta glucan: Health benefits in obesity and metabolic syndrome. J. Nutr. Metabol..

[3] Stevenson L., Phillips F., O’Sullivan K., Walton J. (2012). Wheat bran: Its composition and benefits to health, a European perspective. Int. J. Food Sci. Nutr..

[4] Chen J., Raymond K. (2008). Beta-glucans in the treatment of diabetes and associated cardiovascular risks. Vasc. Health Risk Manag..

[5] Chen J., Seviour R.J. (2007). Medicinal importance of fungal β-(1→3);(1→6)-glucans. Mycol. Res..

[6] Serviour R.J., McNeil B., Fazenda M.L., Harvey L.M. (2011). Operating bioreactors for microbial exopolysaccharide production. Crit. Rev. Biotechnol..

[7] Bohn J.A., BeMiller J.N. (1995). (1→3)-β-d-Glucans as biological response modifiers: A review of structure-functional activity relationships. Carbohydr. Polym..

[8] Soltanian S., Stuyven E., Cox E., Sorgeloos P., Bossier P. (2009). Beta-glucans as immunostimulant in vertebrates and invertebrates. Crit. Rev. Microbiol..

[9] Miranda C.C.B.O., Dekker R.F.H., Serpeloni J.M., Fonseca E.A.I., Colus I.S., Barbosa A.M. (2008). Anticlastogenic activity exhibited by botryosphaeran, a new exopolysaccharide produced by *Botryosphaeria rhodina* MAMB-05. Int. J. Biol. Macromol..

[10] Kiho T., Morimoto H., Sakushima M., Usui S., Ukai S. (1995). Polysaccharides in fungi. XXXV. Anti diabetic activity of an acidic polysaccharide from the fruiting bodies of *Tremella aurantia*. Biol. Pharm. Bull..

[11] Nicolosi R., Bell S.J., Bistrian B.R., Greenberg I., Forse R.A., Blackburn G.L. (1999). Plasma lipid changes after supplementation with beta-glucan fiber from yeast. Am. J. Clin. Nutr..

[12] Hwang H.J., Kim S.W., Lim J.M., Joo J.H., Kim H.O., Kim H.M., Yun J.W. (2005). Hypoglycemic effect of crude exopolysaccharides produced by a medicinal mushroom *Phellinus baumii* in streptozotocin-induced diabetic rats. Life Sci..

[13] Miranda-Nantes C.C.B.O., Fonseca E.A.I., Zaia C.T.B.V., Dekker R.F.H., Khaper N., Castro I.A., Barbosa A.M. (2011). Hypoglycemic and hypocholesterolemic effects of botryosphaeran from *Botryosphaeria rhodina* MAMB-05 in diabetes-induced and hyperlipidemia conditions in rats. Mycobiology.

[14] Narui T., Sawada K., Culberson C.F., Culberson W.L., Shibata S. (1999). Pustulan-type polysaccharides as a constant character of the umbilicariaceae (Lichenized Ascomycotina). Bryologist.

[15] Sassaki G.L., Ferreira J.C., Glienke-Branco C., Torri G., Toni F.D., Gorin P.A.J., Iacomini M. (2002). Pustulan and branched β-galactofuranan from the phytopathogenic fungus *Guignardia citricarpa*, excreted from media containing glucose and sucrose. Carbohydr. Polym..

[16] Vasconcelos A.F., Monteiro N., Dekker R.F.H., Barbosa A.M., Carbonero E., Silveira J.L., Sassaki G.L., Silva R., Conradi da Silva M.L. (2008). Three exopolysaccharides of the β-(1→6)-d-glucan type and a β-(1→3;1→6)-d-glucan produced by strains of *Botryosphaeria rhodina* isolated from rotting tropical fruit. Carbohydr. Res..

[17] Cunha M.A.A., Túrmina J.A., Ivanov R.C., Barroso R.R., Marques P.T., Fonseca E.A.I., Fortes Z.B., Dekker R.F.H., Khaper N., Barbosa A.M. (2012). Lasiodiplodan, an exocellular β-(1→6)-d-glucan from *Lasiodiplodia theobromae* MMPI: Production on glucose, fermentation kinetics, rheology and anti-proliferative activity. J. Ind. Microbiol. Biotechnol..

[18] Delaney B., Carlson T., Frazer S., Zheng T., Hess R., Ostergren K., Kierzek K., Haworth J., Knutson N., Junker K. (2003). Evaluation of the toxicity of concentrated barley β-glucan in a 28-day feeding study in Wistar rats. Food Chem. Toxicol..

[19] Kiho T., Hui J., Yamane A., Ukai S. (1993). Polysaccharides in fungi. XXXII. Hypoglycemic activity and chemical properties of a polysaccharide from the cultural mycelium of *Cordyceps sinensis*. Biol. Pharm. Bull..

[20] Shah S.K., Walker P.A.M.D., Moore-Olufemi S.D., Sundaresan A., Kulkarni A.D., Andrassy R.J. (2011). An Evidence-Based Review of a *Lentinula edodes* Mushroom Extract as Complementary Therapy in the Surgical Oncology Patient. J. Parenteral Enteral. Nutr..

[21] Kim Y.W., Kim K.H., Choi H.J., Lee D.S. (2005). Anti-diabetic activity of β-glucans and their enzymatically hydrolyzed oligosaccharides from *Agaricus blazei*. Biotechnol. Lett..

[22] Wauthier V., Waxman D.J. (2008). Sex-specific early growth hormone response genes in rat liver. Mol. Endocrinol..

[23] Wang L., Behr S.R., Newman R.K., Newman C.W. (1997). Comparative cholesterol-lowering effects of barley β-glucan and barley oil in golden Syrian hamsters. Nutr. Res..

[24] Lorenzi T.F. (2006). Manual Haematology.

[25] Gulye J.V., Camicas J.Z., Diouf A.M. (1988). Ticks and blood parasites in Senegal (*Sahlian zone*). Rev. Elevage Med. Vet. Pays Trop..

[26] Dacie J.V., Lewis S.M. (1991). Practical Haematology.

